# Causal role of lateral prefrontal cortex in mental effort and fatigue

**DOI:** 10.1002/hbm.25146

**Published:** 2020-07-25

**Authors:** Alexander Soutschek, Philippe N. Tobler

**Affiliations:** ^1^ Department for Psychology Ludwig Maximilian University Munich Munich Germany; ^2^ Zurich Center for Neuroeconomics, Department of Economics University of Zurich Zurich Switzerland; ^3^ Neuroscience Center Zurich University of Zurich, Swiss Federal Institute of Technology Zurich Zurich Switzerland

**Keywords:** cognitive effort, decision making, effort discounting, motivation, theta‐burst stimulation

## Abstract

Dorsolateral prefrontal cortex (DLPFC) is well‐known for its role in exerting mental work, however the contribution of DLPFC for deciding whether or not to engage in effort remains unknown. Here, we assessed the causal role of DLPFC in effort‐based decision making. We disrupted functioning of DLPFC with noninvasive brain stimulation before participants repeatedly decided whether to exert mental effort in a working memory task. We found the same DLPFC subregion involved in mental effort exertion to influence also effort‐based decisions: First, it enhanced effort discounting, suggesting that DLPFC may signal the capacity to successfully deal with effort demands. Second, a novel computational model integrating the costs of enduring effort into the effort‐based decision process revealed that DLPFC disruption reduced fatigue after accumulated effort exertion, linking DLPFC activation with fatigue. Together, our findings indicate that in effort‐based decisions DLPFC represents the capacity to exert mental effort and the updating of this information with enduring time‐on‐task, informing theoretical accounts on the role of DLPFC in the motivation to exert mental effort and the fatigue arising from it.

## Graphical abstract

We assessed the causal role of DLPFC in both nonfatigued and fatigued effort‐based decision making. Our findings indicate that in effort‐based decisions DLPFC represents the capacity to exert mental effort as well as the updating of this information with enduring time‐on‐task.

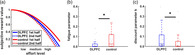

## 1 INTRODUCTION

Remember your toughest academic exam? When you answered the questions you probably engaged fully, thinking of nothing else than the task at hand. Moreover, it may have left you feeling more exhausted than an easier exam. Thus, we seem to experience mental work as effortful. In fact, humans often disengage from demanding mental activity despite its beneficial consequences (Shenhav et al., 2017). However, it remains unclear how the costs of mental effort are computed, as theoretical accounts disagree on why mental work is perceived as effortful. The *resource hypothesis* proposes that subjective effort scales with the degree to which a limited resource is used (Boksem & Tops, 2008), whereas the *cost hypothesis* claims that subjective effort scales with the incurred costs of the exerted effort (Kool & Botvinick, 2014, 2018).

Dorsolateral prefrontal cortex (DLPFC) canonically plays a central role in exerting mental effort (Braver et al., 1997; Miller & Cohen, 2001) and is hypothesized to contribute also to effort‐based decision making in communication with other regions such as the dorsal anterior cingulate cortex (dACC) (Domenech, Redouté, Koechlin, & Dreher, 2018; Shenhav et al., 2017; Shenhav, Botvinick, & Cohen, 2013; Vassena, Deraeve, & Alexander, 2017). While theoretical accounts posit the dACC to trade‐off the value of potential rewards against the required costs (Shenhav et al., 2013; Vassena et al., 2017), the role of DLPFC for motivating engagement in rewarded mental effort remains less well understood. Previous studies reported enhanced DLPFC activation in anticipation of high mental effort demands (Vassena et al., 2014; Vassena, Gerrits, Demanet, Verguts, & Siugzdaite, 2019). However, due to the correlative nature of imaging results, the increased DLPFC activation in these studies could be interpreted either as anticipation of error likelihood and effort costs (Alexander & Brown, 2015; Vassena et al., 2017), or as active preparation for effort exertion (Verguts, Vassena, & Silvetti, 2015).

Crucially, from these alternative interpretations, we can derive dissociable predictions on how the DLPFC causally affects decisions to engage in effort. If enhanced DLPFC activation signals predicted effort costs (which might be forwarded to dACC to integrate effort costs with rewards at stake), experimental disruption of DLPFC activity may reduce the perceived costs of exerting effort, which in turn should result in weaker discounting of reward value by increasing effort requirements (*cost hypothesis*). This hypothesis is supported by previous findings that DLPFC activation during a task switching paradigm predicts the desire to avoid performing further task blocks (McGuire & Botvinick, 2010). Conversely, if DLPFC activation signals the capacity to successfully exert effort, experimental disruption of DLPFC activity should reduce the perceived available mental resources, thereby enhancing effort discounting (*resource hypothesis*). This is because lower mental resources may inform other regions like dACC that current effort demands outweigh potential benefits. Thus, in direct opposition to the cost hypothesis predicting DLPFC downregulation to reduce mental effort discounting due to reduced costs, the resource hypothesis predicts increased mental effort discounting due to downregulated capacity. The current study aimed to dissociate these conflicting accounts by testing how disrupting DLPFC functioning with noninvasive stimulation changes the motivation to exert effort.

DLPFC has also been related to the feeling of fatigue, which is characterized by a lower willingness to engage in effort as a consequence of enduring exertion of high mental effort (Muller & Apps, 2019). Motivational fatigue after enduring work was reported to result in lower DLPFC activation, which in turn reduces the capacity to exert cognitive control (Blain et al., 2019; Blain, Hollard, & Pessiglione, 2016). The lower DLPFC activation after task performance was speculated to reflect a functional adaptation to the costs of exerting control (Hockey & Hockey, 2013; Kurzban, Duckworth, Kable, & Myers, 2013) and may signal to other regions like dACC that the costs of effort exertion are greater than its benefits. Thus, consistent with the possible role of DLPFC for signaling mental resources in effort‐based decision making (as discussed above), motivational fatigue may reflect an updating of the information about available, DLPFC‐implemented, control capacities. However, also the evidence regarding the relationship between DLPFC and fatigue is only correlative to date. A further goal of the current study therefore was to assess the causal role of DLPFC for updating the ability to exert mental effort with enduring time‐on‐task.

Here, we employed continuous theta‐burst stimulation (cTBS) and computational modeling to dissociate between competing theoretical accounts of the role of DLPFC in motivating strenuous mental work. Introducing a novel experimental paradigm that allows considering the impact of fatigue on motivation for mental effort, we tested how mental effort exertion with perturbed DLPFC function changes the nonfatigued and fatigued motivation to engage in mental work. While formal models describing the discounting of reward value as function of effort costs are widely used in the literature (Chong et al., 2017; Hartmann et al., 2015; Hartmann, Hager, Tobler, & Kaiser, 2013; Soutschek et al., 2020), formal models incorporating the impact of fatigue into value computations (Muller & Apps, 2019) have not been tested empirically so far. To test how DLPFC cTBS influences both fatigued and nonfatigued effort discounting, we therefore incorporated possible influences of fatigue into existing formal models of effort discounting. Disentangling nonfatigued and fatigued effort discounting via experimental design and formal modeling allowed us to test the following research questions: First, we assessed whether DLPFC disruption leads to either stronger (resource hypothesis) or weaker (cost hypothesis) nonfatigued effort discounting. Second, we tested whether DLPFC cTBS reduces the impact of fatigue on effort‐based decision making by disrupting the updating of DLPFC‐implemented mental resources with increasing fatigue.

## 2 MATERIALS AND METHODS

### 2.1 Participants

Sixty volunteers (29 female, M_age_ = 23.7 years, SD_age_ = 2.7 years) were randomly assigned to one of the two stimulation groups (DLPFC or vertex). Power calculations based on a previous study (Mottaghy et al., 2003) suggested that a minimum of 25 participants per stimulation group was needed to replicate the significant effects of DLPFC stimulation on N‐back performance with a power of 80% (alpha = 5%, one‐tailed). All volunteers gave written informed consent. The study protocol was approved by the Research Ethics Committee of the canton of Zurich. Participants received 60 Swiss francs for their participation and a monetary bonus that depended on their choices (see below).

### 2.2 Stimuli and task design

#### 2.2.1 N‐back task

Participants performed a letter version of the N‐back task (Braver et al., 1997) in which they were presented a stream of letters (b, c, d, g, p, t, and w). We instructed participants to press the space bar on a keyboard if the currently presented letter was a target stimulus, that is, if it was identical to the letter presented N trials before. Thus, participants permanently maintained and updated N letters in working memory. Each trial started with the presentation of a letter (500 ms), followed by a fixation cross (1,500 ms). If the current letter was a target, the response had to be executed before the start of the next trial. Previous research suggests that humans perceive this task as mentally demanding and that the value of performing the task decreases with increasing N (Westbrook, Kester, & Braver, 2013). To calibrate the difficulty of the N‐back task to individual performance levels (see below), participants performed one block of the 2‐back, 3‐back, 4‐back, and 5‐back condition (each block lasting 2 min) at the start of the experiment. If performance in the 5‐back condition was ≥40% correct, they additionally performed a 6‐back block (where no participant reached 40% correct).

#### 2.2.2 Effort‐related decision task

**FIGURE 1:**
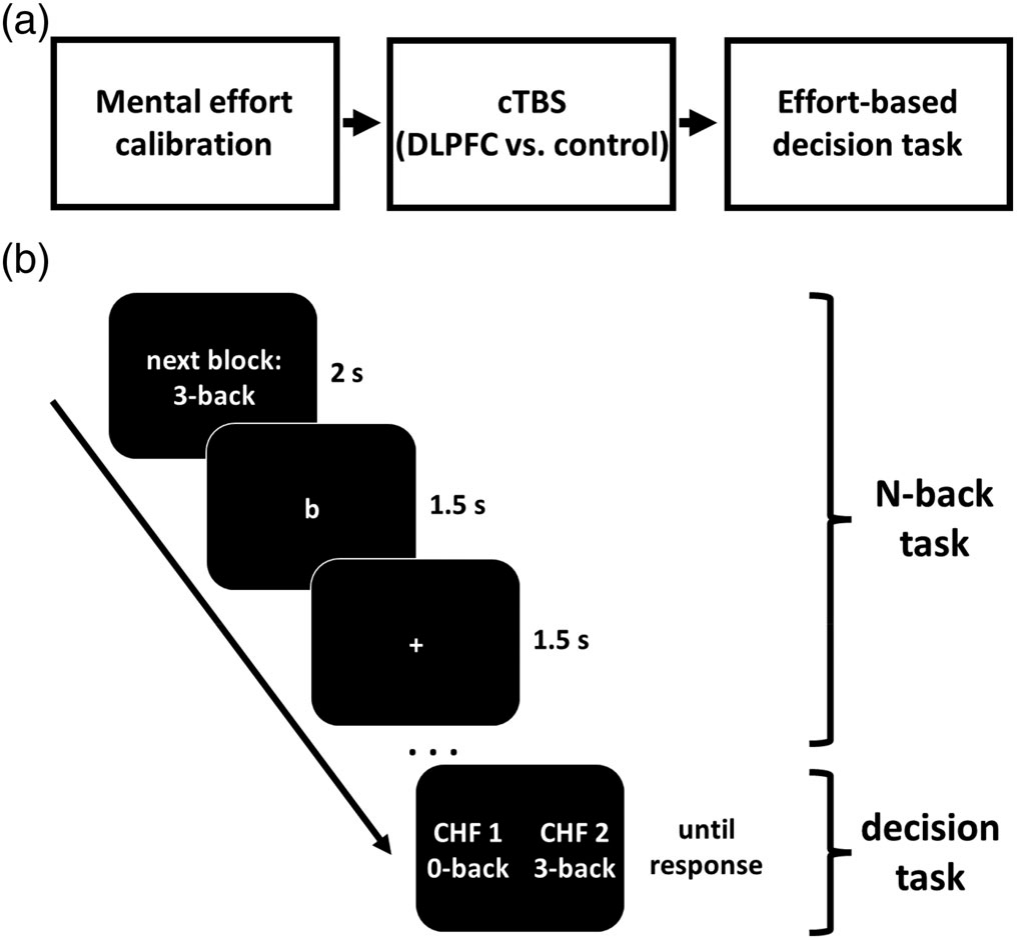
Task procedure and example trial. (a) Participants first performed the N‐back task to determine their maximum effort level (N_max40%_, i.e., the highest N for which accuracy was at least 40%). Then participants received cTBS to either DLPFC or vertex as control site, and performed the effort‐based decision task. (b) Before making effort‐based decisions at a specific effort level, participants performed 10 trials (duration = 30 s) of the N‐back task at that same effort level, allowing participants to accumulate effort and base upcoming choices on actual experience. Subsequently, participants decided between performing the given N‐back level for a monetary reward (1.1–2.5 Swiss francs; effortful reward option) and passively viewing letters of the N‐back task for 1 Swiss franc (effort‐free reward option). Next, a different 30 s N‐back block with the effort level of the subsequent effortful choice option started, and participants again decided between the effortful and the effort‐free reward options. Only at the end of the experiment (i.e., after participants had completed all choices), one choice was randomly selected and implemented. This ensured that effort requirements during the decision task were independent of the actual choices participants made

In each trial, participants decided between two choice options (Figure 1). For the effort‐free option, participants obtained 1 Swiss franc without having to perform the N‐back task. In contrast, the effortful option required participants to perform low, medium, or high levels of mental effort in order to obtain a monetary reward (1.1–2.5 Swiss francs). The required mental effort was calibrated to an individual's performance level in the N‐back task (N_max40%_, i.e., the highest N at which a participant showed ≥40% correct responses). For example, if N_max40%_ = 3, the low, medium, and high effort levels corresponded to the 2‐back (N_max40%_ − 1), 3‐back (N_max40%_), and 4‐back (N_max40%_ + 1) conditions, respectively. Before each decision, participants performed a miniblock of the corresponding N‐back condition with 10 trials for 30 s. This allowed participants to experience and update the strain required by the effortful option of each choice. Thereby, we could assess the impact of accumulated effort exertion on effort‐based choice.

At the end of the experiment, one trial was randomly determined and implemented. If participants had chosen the effortful option, they had to perform the corresponding level of the N‐back task for 2 min with a rate of correct responses ≥25% to obtain the reward; if performance was below 25%, they obtained 1 Swiss franc (as for the effort‐free option, allowing to control for stimulation effects on risk preferences). Participants were not informed about the exact performance threshold; instead, they were instructed that they would win the bonus as long as they worked hard for it. Moreover, they were informed that if the data suggested that they had not invested sufficient effort they would receive no bonus (Westbrook et al., 2013). This procedure ensures that choices are guided by participants' effort preferences rather than by the perceived risk of not obtaining the bonus despite strong task engagement. To disentangle effort from time preferences, participants knew that they had to passively view the letters for the N‐back task for 2 min if they had chosen the effort‐free option. This ensured that they did not choose the effort‐free option to finish the experiment earlier.

#### 2.2.3 Rating tasks

To measure the impact of cTBS on fatigue, we administered a rating task where participants (still under the impact of cTBS) indicated on a 20‐point scale their level of fatigue (1 = not tired; 20 = very tired) after the decision task. In addition, participants rated their mood. Finally, to assess potential cTBS effects on risk preference, they made a choice between a risk‐free (1 Swiss franc for sure) and a risky reward (50% chance of winning either 2 or 0 Swiss francs).

#### 2.2.4 Questionnaires

Before receiving cTBS, participants filled in questionnaires measuring baseline reward sensitivity and motivation traits. The Snaith–Hamilton Pleasure Scale (Snaith et al., 1995) measures individual differences in anhedonia, whereas the behavioral inhibition/activation system (BIS/BAS [Carver & White, 1994]) questionnaire represents a standard measure of individual reward sensitivity.

### 2.3 Procedure

Participants first filled in the LARS and BIS/BAS questionnaires as baseline measures of reward sensitivity. After motor threshold determination, participants performed the N‐back task to determine their maximum effort level (N_max40%_). Following cTBS to either DLPFC or vertex, they made 24 choices in the effort‐based decision task. Before each decision, participants had to perform the N‐back task with the effort level of the subsequent effortful reward option for 30 s. Finally, participants performed the rating tasks while still under the influence of cTBS. At the very end of the experiment, one trial was randomly selected and implemented as bonus trial.

### 2.4 cTBS

Participants were stimulated either over the left DLPFC (30 participants) or over the vertex (30 participants) with a standard cTBS protocol (Huang, Edwards, Rounis, Bhatia, & Rothwell, 2005). We used a Super Rapid stimulator (Magstim Co.) and a figure‐of‐eight coil (internal diameter of 7 cm). For cTBS, bursts of 3 stimuli at 50 Hz were repeated with a frequency of 5 Hz for 40 s (600 pulses in total) at 80% of the active motor threshold. Motor threshold corresponded to the lowest pulse intensity required to elicit a motor‐evoked potential larger than 200 μV from the contralateral first dorsal interosseous muscle on more than five out of 10 trials while the participant maintained a contraction of 20% maximum force. This cTBS protocol reduces the excitability of the stimulated brain region for up to 60 min (Huang et al., 2005).

We determined stimulation sites using individual T1‐weighted structural scans and Brainsight frameless stereotaxy (Rogue Research). For the DLPFC site, we used coordinates for the left DLPFC (MNI coordinates: *x* = −41, *y* = 27, *z* = 25) from a meta‐analysis on the N‐back task (Owen, McMillan, Laird, & Bullmore, 2005). For each participant, we transformed the DLPFC coordinates into the native space of their structural scan. As control site, we used the vertex (meeting point of the pre‐ and postcentral sulcus in the interhemispheric fissure). The cTBS coil was positioned tangentially to the cortical surface over these sites during stimulation, with the handle pointing backward. For DLPFC cTBS, the handle was angled 45° away from the midline (Mottaghy et al., 2003).

### 2.5 Data analysis

The statistical analysis of the behavioral data was performed with Matlab R2016b (MathWorks, Natick, MA) and IBM SPSS Statistics 22. For the effort‐based decision task, we computed a mixed generalized linear model (MGLM) to analyze dummy‐coded choices (0 = effort‐free option, 1 = effortful option). The MGLM included the following fixed‐effects predictors: cTBS (0 = vertex, 1 = DLPFC), Trial (trial 1–24; recoded to the range of [0;1]), Effort level (*z*‐standardized), Reward magnitude (*z*‐standardized), and the interaction terms modeling the impact of cTBS and Trial on Effort level and Reward magnitude. We additionally modeled participant‐specific random intercepts and all within‐subject effects (Effort level, Reward magnitude, Trial, Trial × Effort level, Trial × Reward magnitude) as random slopes. In the N‐back task, we regressed log‐transformed reaction times (RTs) on fixed‐effects predictors for cTBS, Effort level, Trial, and all interaction terms. As random effects, we entered random intercepts as well as random slopes for Effort level, Trial, and the interaction term. Degrees of freedom were computed using the Satterthwaite approximation. In addition, we computed Bayes factors as indicators of how strongly the data favor the alternative over the null hypothesis (BF_10_) with the brms package in R.

We analyzed choice behavior also in a model‐based fashion. Note that models of effort discounting disagree on whether the devaluation of subjective reward value by increasing (mental) effort is best described with linear, hyperbolic, or parabolic functions (Bialaszek, Marcowski, & Ostaszewski, 2017; Chong et al., 2017; Hartmann et al., 2013; Soutschek et al., 2020; Westbrook et al., 2013). We therefore modeled choices in the decision task with the following discount functions (Equations (1), (2), (3)):

(1) $${\mathrm{SV}}_{\mathrm{eff}}=\text{reward}-k\times {\text{effort}}_{\text{current}}\left(\text{linear}\right)$$

(2) $${\mathrm{SV}}_{\mathrm{eff}}=\frac{\text{reward}}{1+k\times {\text{effort}}_{\text{current}}}\left(\text{hyperbolic}\right)$$

(3) $${\mathrm{SV}}_{\mathrm{eff}}=\text{reward}-k\times {\text{effort}}_{\text{current}}^{2}\left(\text{parabolic}\right)$$

where *SV*_eff_ represents the subjective value of the effortful reward option and *k* indicates the individual degree of effort discounting. To translate subjective values (as given by Equations (1), (2), (3)) into choice, we used a standard softmax function (Equation 4).

(4) $$P\left(\text{choice of effortful option}\right)=\frac{1}{1+\exp(-\beta_{\text{temp}\times \left({\mathrm{SV}}_{\mathrm{eff}}-1\right)})}$$

This function captures the likelihood of choosing the effortful reward option as a function of the value difference (multiplied by the inverse temperature parameter *β*_temp_) between the effortful reward option (*SV*_eff_) and the effort‐free option (which was fixed to 1 Swiss franc). Consistent with previous studies (Chong et al., 2017; Hartmann et al., 2013), a parabolic discounting model explained the data better (*R* ^2^ = .68) than a hyperbolic (*R* ^2^ = .63) or linear model (*R* ^2^ = .62).

Finally, we tested how fatigue affects the computation of effort costs. We considered four alternatives how the accumulated amount of effort might affect parabolic effort discounting (which explained choices better than linear or hyperbolic discounting; see above): First, we assumed that a fatigue parameter φ might be either added to, or multiplied with, the discount factor *k*. Second, we tested whether accumulated effort either linearly or nonlinearly (log‐transformed) affects effort discounting. We thus compared the following four alternative models:

Model 1: ${\mathrm{SV}}_{\mathrm{eff}}=\text{reward}-\left(k\times \varphi \times {\text{effort}}_{\text{accumulated}}\right)\times {\text{effort}}_{\text{current}}^{2.}$

Model 2: ${\mathrm{SV}}_{\mathrm{eff}}=\text{reward}-\left(k+\varphi \times {\text{effort}}_{\text{accumulated}}\right)\times {\text{effort}}_{\text{current}}^{2}$

Model 3: ${\mathrm{SV}}_{\mathrm{eff}}=\text{reward}-\left(k\times \varphi \times \log\left(1+{\text{effort}}_{\text{accumulated}}\right)\right)\times {\text{effort}}_{\text{current}}^{2}$

Model 4: ${\mathrm{SV}}_{\mathrm{eff}}=\text{reward}-\left(k+\varphi \times \log\left(1+{\text{effort}}_{\text{accumulated}}\right)\right)\times {\text{effort}}_{\text{current}}^{2}$

In Models 3 and 4, we added 1 to the amount of accumulated effort (sum of performed n‐back levels) to avoid negative values after the log‐transformation in cases of effort_accumulated_ = 0. Model comparisons revealed that Model 4 (using an additive link between *k* and *φ* as well as log‐transformed effort costs) explained the observed data better (*R* ^2^ = .703) than all other models (Model 1: *R* ^2^ = .592; Model 2; *R* ^2^ = .696; Model 3: *R* ^2^ = .659). In addition, this model explained the observed data better (*R* ^2^ = .70) than the standard parabolic model (*R* ^2^ = .68).

In all model‐based analyses, parameters were estimated using maximum likelihood methods as implemented in Matlab. Because the resulting individual parameter estimates were not normally distributed, we used nonparametric ordinal regressions implemented in SPSS to assess statistical significance.

## 3 RESULTS

### 3.1 Baseline measures

The two stimulation groups were balanced with regard to baseline measures of anhedonia (Snaith et al., 1995), *t*(58) = 1.37, *p* = .18, or reward sensitivity (behavioral inhibition/activation system, BIS/BAS [Carver & White, 1994]), BIS: *t*(58) = 0.00, *p* = 1, BAS: *t*(58) < 1, *p* = .78. Also *N*_max40%_ did not significantly differ between stimulation groups, *χ* ^*2*^ = 5.57, *p* = .23. Any cTBS effects on N‐back performance or decision making thus cannot be explained by pre‐existing baseline differences in these measures.

### 3.2 DLPFC cTBS impairs mental effort exertion

**FIGURE 2:**
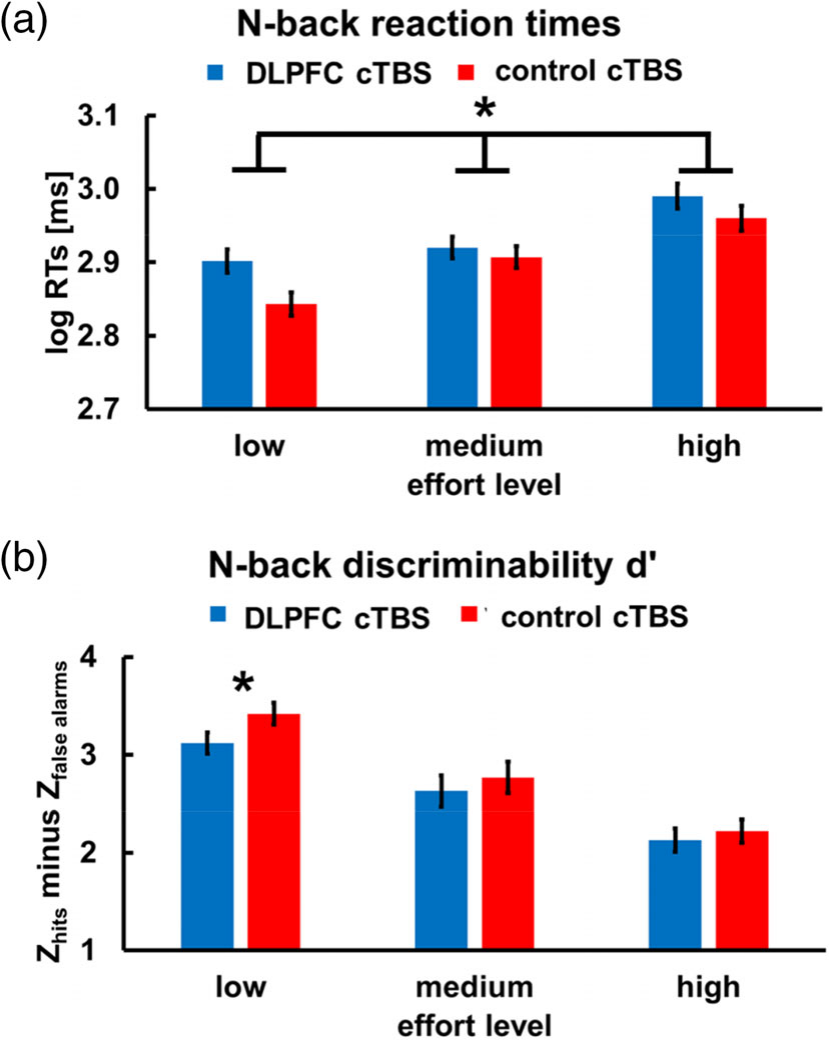
Effects of downregulating DLPFC activity on mental effort performance. DLPFC cTBS, relative to control cTBS, increased (a) log‐transformed RTs in the N‐back task and (b) tended to reduce discriminability d' in the low effort condition. These data replicate previous findings of causal DLPFC involvement in mental effort exertion (Mottaghy et al., 2003; Oliveri et al., 2001; Sandrini et al., 2008). Error bars indicate standard error of the mean. Asterisks indicate significant effects (*p < .05)

First, we tested whether we replicate previous reports of a causal involvement of DLPFC in mental effort *exertion* (Mottaghy et al., 2003; Oliveri et al., 2001; Sandrini, Rossini, & Miniussi, 2008). An MGLM on log‐RTs in the N‐back task revealed a significant effect of cTBS, *β* = .05, *t*(132) = 2.14, *p* = .03, BI_10_ = 1.7, indicating slower RTs following DLPFC compared with control cTBS (Figure 2a and Table 1). We observed no interactions between cTBS and Effort level or Trial, all *β* < .05, all *t* < 1.73, all *p* > .09, all BI_10_ < 1.2. The main effect of Effort level was not significant, *β* = .03, *t*(229) = 1.48, *p* = .14, BI_10_ = 2.6, but higher effort levels resulted in performance decrements with increasing time‐on‐task, *β* = .05, *t*(1798) = 2.06, *p* = .04, BI_10_ = 1.9. Taken together, perturbation of DLPFC slowed responding during mental effort *exertion*.

**TABLE 1:** Results of MGLM assessing log‐transformed reaction times in the N‐back task

	Beta	*t*‐value	*Df*	*p*	BF_10_
Intercept	2.90 (0.02)	188.39	132	<.001	
cTBS	0.05 (0.02)	2.14	132	.03	1.7
Effort	0.03 (0.02)	1.48	229	.14	2.6
Trial	0.02 (0.02)	1.09	128	.28	0.4
cTBS × effort	0.03 (0.02)	1.37	229	.17	0.6
cTBS × trial	−0.05 (0.03)	1.72	131	.09	1.1
Effort × trial	0.05 (0.02)	2.06	1,798	.04	1.9
cTBS × effort × trial	−0.05 (0.03)	1.61	1,798	.11	0.9

*Note:* Standard errors of the mean are reported in brackets. BF_10_ is the Bayes factor indicating how strongly the data favor the alternative over the null hypothesis (note that no Bayes factor can be computed for fixed‐effect intercepts in mixed models).

Furthermore, we tested for stimulation effects on N‐back target detection (controlling for false alarms using discriminability d'). An ANCOVA showed no effect of cTBS or a cTBS × Effort level interaction, both *F* < 1.53, *p* > .22, both partial eta^2^ < 0.026, both BF_10_ < 1.1. However, when we explored cTBS effects for each effort level separately, we observed that DLPFC cTBS tended to impair performance in low‐effort blocks, *t*(57) = 1.87, *p* = .06, BF_10_ = 2.3, but not in medium‐ or high‐effort blocks, both *t*(57) < 1, both *p* > .54, both BF_10_ < .6 (Figure 2b). This may reflect floor effects (i.e., cTBS cannot further reduce already low discriminability at higher effort levels). In any case, the RT and discriminability findings corroborate previous reports of a causal involvement of DLPFC in exerting mental effort.

**TABLE 2:** Results of MGLM assessing binary choice in the effort‐based decision task

	Beta	*t*‐value	*Df*	*p*	BF_10_
Intercept	2.00 (0.56)	3.62	74	.001	
cTBS	−0.54 (0.78)	0.70	74	.49	0.5
Effort	−1.57 (0.42)	3.70	135	<.001	120.5
Reward	1.31 (0.40)	3.28	397	.001	1,427
Trial	−0.55 (0.52)	1.07	1,428	.29	0.3
cTBS × effort	−1.28 (0.61)	2.09	161	.04	3.0
cTBS × reward	0.41 (0.56)	0.72	396	.47	0.3
cTBS × trial	0.69 (0.73)	0.96	1,428	.34	0.6
Effort × trial	−0.79 (0.73)	1.09	151	.28	0.6
Reward × trial	0.30 (0.60)	0.51	1,428	.61	0.4
cTBS × effort × trial	2.40 (1.01)	2.38	143	.02	4.8
cTBS × reward × trial	0.04 (0.84)	0.05	1,428	.96	0.4

*Note:* Standard errors of the mean are reported in brackets. BF_10_ is the Bayes factor indicating how strongly the data favor the alternative over the null hypothesis (note that no Bayes factor can be computed for fixed‐effect intercepts in mixed models).

### 3.3 DLPFC cTBS reduces fatigue after effort exertion

**FIGURE 3:**
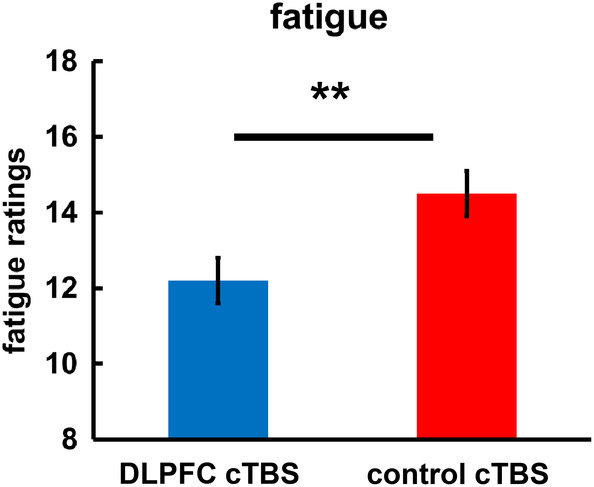
Effects of downregulating DLPFC activity on self‐reported fatigue. DLPFC cTBS, relative to control cTBS, reduced self‐reported feelings of fatigue measured after task performance. Error bars indicate standard error of the mean. Asterisks indicate significant effects (**p < .01)

After the decision task (still under the impact of cTBS), we measured mood, risk preferences, and subjective fatigue. We observed no cTBS‐induced changes in mood or risk preferences, both *t* < 1.15, *p* > .25. Importantly, we observed that participants felt less exhausted at the end of the experiment (i.e., after the decision task) after DLPFC than after control cTBS, *t*(58) = 2.78, *p* = .008, BF_10_ = 81.5 (Figure 3; we note, though, that our study included no baseline measure of fatigue before effort exertion). There were no significant differences in time of day when participants of the DLPFC and control cTBS groups started the experiment, *χ* ^*2*^(9) = 11.53, *p* = .24, suggesting that the stimulation effects on fatigue could not be explained by the DLPFC cTBS group performing the experiment later in the day than the control group. Thus, impairing mental effort exertion did not result in compensatory, more fatiguing mental work. Instead, it reduced the sensation of work‐related exhaustion.

### 3.4 DLPFC cTBS changes willingness to engage in effort with time‐on‐task

Next, we tested whether DLPFC affects not only the sensation of fatigue after mental effort *exertion* but also *decisions* to engage in effort. We computed an MGLM that regressed binary choices of effortful versus effort‐free rewards on predictors for cTBS, Effort level, Reward magnitude, Trial, and the interaction effects. Because the predictor Trial ranged from 1 to 24, the lower‐level effects without the predictor Trial describe behavior before effort accumulation (i.e., Trial = 0). Choices of the effortful option decreased with increasing effort, *β* = −1.57, *t*(135) = 3.70, *p* < .001, BF_10_ = 120.5, and increased with reward magnitude, *β* = 1.31, *t*(397) = 3.28, *p* = .001, BF_10_ = 1,427.0. Importantly, before effort accumulation (i.e., at the start of the decision task, where the value of the predictor Trial was set to the reference category 0) DLPFC disruption resulted in stronger effort discounting than control stimulation, cTBS × Effort level, *β* = −1.28, *t*(161) = 2.09, *p* = .04, BF_10_ = 3.0. This reduced willingness to engage in mental work is consistent with the resource hypothesis. cTBS did not modulate the impact of reward magnitude, *β* = .41, *t* < 1, *p* = .47, BF_10_ = 0.3, and cTBS effects were significantly stronger on effort than on reward, *Z* = 2.04, *p* = .04. Thus, cTBS affected specifically the impact of effort on choices, at variance with domain‐general cTBS effects on decision making.

**FIGURE 4:**
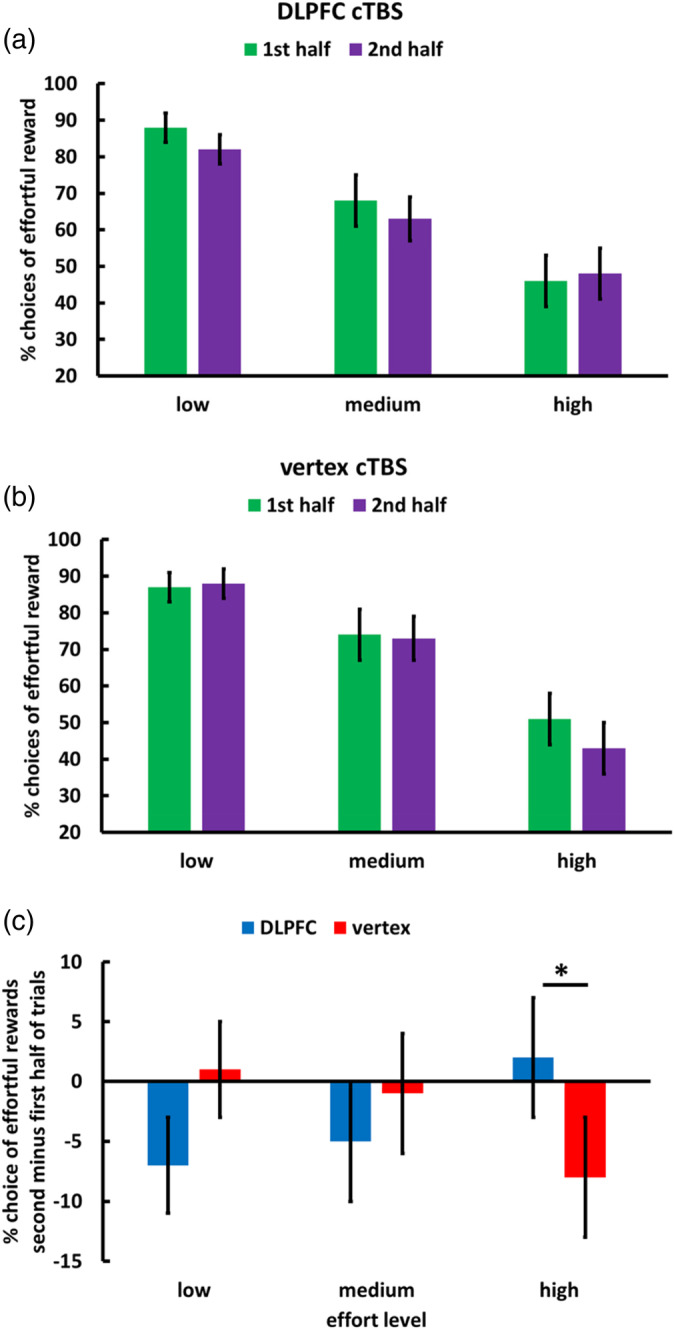
Effects of downregulating DLPFC activity on motivation to exert mental effort. (a) DLPFC disruption prevented increasing effort discounting with increasing time‐on‐task. Conversely (b), the control group showed steeper effort discounting toward the end of the experiment, consistent with the assumption that fatigue increases effort discounting. (c) The impact of time‐on‐task on willingness to accept the high effort option (indicating increased effort discounting) was significantly stronger after control compared with DLPFC cTBS. Please note that participants had to exert the chosen effort not immediately after each choice but only for one randomly selected decision at the end of the experiment, such that stimulation effects on choices cannot be explained by differences in the amount of effort exerted during the decision task. For illustration, we plot data separately for the first and second half of trials. Error bars indicate standard error of the mean. Asterisks indicate significant effects (*p < .05)

We also observed that the impact of cTBS on effort discounting depended on accumulated effort, cTBS × Effort level × Trial, *β* = 2.40, *t*(143) = 2.38, *p* = .02, BF_10_ = 4.8 (Figure 4 and Table 2), while the cTBS × Reward × Trial interaction was not significant, *β* = .04, *t*(1428) = 0.05, *p* = .96, BF_10_ = 0.4. We note, though, that a direct comparison of the regression weights for the cTBS × Effort level × Trial and the cTBS × Reward × Trial interactions revealed only a trend‐level effect, *Z* = 1.80, *p* = .07. To resolve the cTBS × Effort level × Trial interaction, we computed separate MGLMs for each effort level: For high effort, but not for medium and low effort, we observed a marginally significant negative effect of trial, *β* = −1.70, *t*(71) = 1.95, *p* = .05, BF_10_ = 3.50, suggesting that in the control group participants were less willing to choose the effortful option with increasing time‐on‐task. This is consistent with stronger effort aversion under increasing fatigue. Importantly, this de‐motivating impact of accumulated effort was significantly reduced after DLPFC cTBS, cTBS × Trial, *β* = 2.73, *t*(66) = 2.31, *p* = .02, BF_10_ = 2.88. We note that the results for the decision task are robust to controlling for individual differences in anhedonia. Thus, DLPFC disruption increased effort discounting before effort accumulation and at the same time reduced the impact of time‐on‐task on effort discounting.

### 3.5 Computational modeling reveals crucial role of DLPFC for accumulating fatigue

To further investigate the role of DLPFC for mental work, we analyzed choice behavior also with a model‐based approach by fitting discount functions to each individual's choices. Consistent with previous studies (Chong et al., 2017; Hartmann et al., 2013), a parabolic discounting model explained the data better (*R* ^2^ = .68) than a hyperbolic (*R* ^2^ = .63) or linear model (*R* ^2^ = .62; see Section 2 for details). The winning parabolic model had the following form:

$${\mathrm{SV}}_{\text{effortful reward}}=\text{reward}-k\times {\text{effort}}_{\text{current}}^{2}$$

As for the model‐free analysis, we also included a term measuring the impact of increasing time‐on‐task on effort discounting to the best‐fitting parabolic model in order to account for effects of fatigue on effort discounting. We considered different possibilities for how fatigue might computationally affect effort discounting: First, based on a recent theoretical account suggesting that fatigue increases effort discounting (indicated by the discount factor *k*; Muller & Apps, 2019), we tested for either additive or multiplicative relationships between effort discounting and performance‐induced fatigue. In addition, we considered the possibility that mental work might either linearly or nonlinearly increase fatigue. In the winning model that explained the data best (see Section 2), a fatigue parameter *φ* is added to the discount factor *k* according to the following formula:

$${\mathrm{SV}}_{\text{effortful reward}}=\text{reward}-\left(k+\varphi \times \log\left(1+{\text{effort}}_{\text{accumulated}}\right)\right)\times {\text{effort}}_{\text{current}}^{2}$$

**FIGURE 5:**
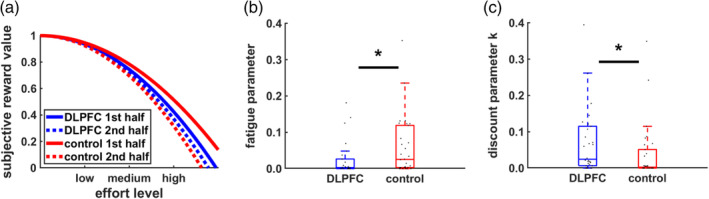
Model‐based fatigue effects on effort discounting. (a) Consistent with the model‐free results, enduring task performance increased effort discounting only in the control cTBS, not the DLPFC cTBS, group. (b) The individually specific impact of fatigue on effort was significantly lower in the DLPFC than the control cTBS group. (c) Compared to vertex disruption, DLPFC disruption increased effort discounting at the start of the experiment before fatigue accumulation, as indicated by significant cTBS effects on the discount parameter k. Asterisks indicate significant effects (*p < .05)

In this model, *k* determines the degree of effort discounting before effort exertion. As exerted effort accumulates, a fatigue parameter *φ* is added to *k* (higher values of *k* imply stronger effort discounting). In other words, if no effort has been exerted so far (effort_accumulated_ = 0), the term “log(1 + effort_accumulated_)” equals zero and thus φ has no impact on effort discounting. With increasing accumulated effort, a larger fatigue term is added to k, resulting in stronger effort discounting. Nonparametric ordinal regressions revealed significant cTBS effects on k, beta = 1.06 *Wald's W*(1) = 5.21, *p* = .02, BF_10_ = 2.9, with DLPFC disruption resulting in stronger effort discounting (at the start of the task before any effort was exerted) than control stimulation. This supports the resource hypothesis according to which reducing resources to exert mental effort should lower the motivation to engage in rewarded effort. Importantly, individual fatigue parameters *φ* were lower in the DLPFC than the control cTBS group, beta = −0.93 *W*(1) = 4.10, *p* = 0.04, BF_10_ = 2.9 (Figure 5). Together with the cTBS effects on fatigue ratings and the model‐free results, these findings provide converging evidence that DLPFC disruption both increases effort discounting and reduces the impact of accumulated effort on motivational fatigue.

If *φ* captures individual differences in fatigue, it should relate to self‐reported fatigue. Consistent with this prediction, *φ* was positively correlated with fatigue ratings at the end of the experiment, Kendall's *τ* = 0.17, *p* = .04, one‐tailed. This correlation was robust to controlling for TMS‐effects on the constituent measures. In contrast, there were no significant correlations between the parameters *φ* and *k*, *τ* = −0.08, *p* = .35, as well as between *k* and self‐reported fatigue, *τ* = 0.02, *p* = .81, suggesting that *k* and *φ* measure dissociable constructs. Moreover, performance in the N‐back task (log‐RTs) predicted the degree of effort discounting *k*, *τ* = 0.30, *p* = .001, but was uncorrelated with *φ*, *τ* = 0.08, *p* = .38. Taken together, our findings are consistent with the assumption that fatigue reduces the motivation to engage in rewarded effort, with DLPFC disruption decreasing the influence of accumulated fatigue on decision making.

To test the relationship between cTBS‐induced performance decrements in the N‐back and stimulation effects on effort discounting and fatigue more thoroughly, we conducted two separate mediation analyses testing whether cTBS effects on effort discounting *k* or fatigue *φ* are mediated by cTBS effects on N‐back performance (log‐RTs). When entering mean log‐RTs in the N‐back task as predictor in the ordinal regression on k, the predictor N‐back performance showed a significant effect, beta = 11.35, *W*(1) = 10.74, *p* = .001, whereas the effect of cTBS now no longer passed the statistical threshold, beta = .85 *W*(1) = 3.38, *p* = .07, in line with a mediation effect. This interpretation was supported by a significant Sobel test (testing the significance of the indirect mediation path), *z* = 1.99, *p* = .047. Together, these data suggest that impairments in the ability to exert mental effort mediate the stronger effort discounting after DLPFC cTBS. In contrast, when conducting an analogous mediation analysis on fatigue, cTBS effects on *φ* remained significant, beta = −1.02, *W*(1) = 1.18, *p* = .03, even when controlling for N‐back performance, and also the Sobel test yielded no significant result, *z* < 1, *p* = .34. Thus, there is no evidence that lower fatigue after DLPFC disruption can be explained by impaired working memory performance.

Last, we also explored the possibility that time‐on‐task affects choice consistency (measured by the inverse temperature parameter *β*_temp_ in Equation 4) rather than effort discounting. For this purpose, we modified our computational model of fatigue effects by adding the fatigue term “*φ* × log(1 + effort_accumulated_)” to the inverse temperature parameter *β*_temp_ instead of to the discount factor *k*. However, this model revealed no significant cTBS effects on either *β*_temp_ or *φ*, both W(1) < 1, both *p* > .64. There was thus no evidence for time‐on‐task effects on choice consistency.

## 4 DISCUSSION

We tested competing hypotheses about the causal function of DLPFC in effort‐based decisions, according to which DLPFC either signals the availability of mental resources (resource hypothesis) or the inherent costliness of mental work (cost hypothesis). First, we found that disrupting DLPFC functioning impairs the motivation for mental work. This conclusion is supported by both the model‐free and model‐based results showing steeper effort discounting with DLPFC stimulation than control stimulation before fatigue accumulation. This appears inconsistent with the hypothesis that DLPFC activation in effort‐based choice signals the costliness of mental work (McGuire & Botvinick, 2010), because according to this view DLPFC should have reduced the perceived costliness of mental work, thereby promoting decisions to engage in effort. Instead, our findings support the resource hypothesis and suggest that individuals decide against engaging in demanding work if they anticipate insufficient resources to perform it after DLPFC perturbation (Boksem, Meijman, & Lorist, 2006; Boksem & Tops, 2008). This interpretation is also supported by the mediation analysis suggesting that stimulation effects on effort discounting can be explained by the impact of cTBS on N‐back performance.

Our results further suggest that DLPFC plays a causal role in mediating the impact of accumulated effort exertion on effort preferences. Strikingly, three distinct measures (model‐free choice data, self‐reported fatigue, and computational model parameters) provide converging evidence that DLPFC disruption reduced fatigue and its impact on effort‐based decision making. Previous studies reported decreased DLPFC activation to correlate with fatigue after enduring effort exertion (Blain et al., 2016; Blain et al., 2019; Ishii, Tanaka, & Watanabe, 2014), and it has been speculated that decreased DLPFC activity after enduring work reflects a functional adaptation to the costs of effort exertion (Hockey & Hockey, 2013; Kurzban et al., 2013). Consistent with our interpretation of cTBS effects on choices before effort accumulation, the decrease in DLPFC activation after enduring effort may signal dACC a reduced capacity to deal with high effort demands, thereby increasing the costs of effortful rewards. DLPFC cTBS may have disrupted this decrease in DLPFC activation with time‐on‐task, which in turn prevented the adaptation of cost–benefit weighting processes to increasing fatigue. Note that the impact of cTBS on fatigue was not mediated by stimulation effects on N‐back performance, which speaks against the possibility that reduced effort exertion per se can explain the lower fatigue after DLPFC cTBS (though a null finding cannot fully rule out this possibility). Moreover, our interpretation does not imply that decreased DLPFC activation represents the physiological processes underlying fatigue or the perceived costs of enduring work. Indeed, these processes are still a matter of controversial debate. For example, accumulation of toxic beta amyloids in neural tissue has been discussed as potential origin of costs (Holroyd, 2016). In any case, even though the proposed interpretation of the cTBS effects on fatigue remains somewhat speculative and may require further investigation, our data provide first evidence for a causal link between task‐related DLPFC activation and performance‐induced feelings of fatigue.

It is worth noting that DLPFC cTBS affected preferentially the processing of effort while reward magnitude remained unchanged. This speaks against the alternative explanation that DLPFC disruption impaired decision making per se, because according to this account cTBS should have affected both effort and reward processing. We note that cTBS effects on effort were significantly stronger than on reward before effort accumulation, whereas cTBS effects on reward and effort as function of trial number showed only a trend‐level difference, such that the specificity of DLPFC for effort relative to reward processing in effort‐based choice needs to be interpreted with caution. Moreover, effort discounting was steeper under DLPFC than control cTBS (before accumulating effort), which is at variance with the possibility that cTBS increased decision noise by distorting effort representations in DLPFC because this should have reduced, instead of increased, the impact of effort on choices.

Our results inform recent neural frameworks of effort discounting. While theoretical models assign dACC a central role in trading off costs against benefits and in recruiting DLPFC if the benefits surpass the costs of control (Kool & Botvinick, 2018; Shenhav et al., 2013; Shenhav et al., 2017), it remained open where the cost signal stems from. Our results ascribe the DLPFC subregion involved in effort exertion also a crucial role in computing the predicted capacity to successfully exert effort, which constitutes a parsimonious solution to the problem of cost calculation. The strength of DLPFC activation could inform dACC about the likelihood of successfully dealing with future demands (Alexander & Brown, 2015; Vassena et al., 2014; Vassena et al., 2019), enabling dACC to compare the required work with the potential benefits.

The hypothesized DLPFC involvement in motivating mental effort engagement has implications for psychiatric disorders involving dysfunctions in motivation or cognitive control. Major depressive disorder, for example, is characterized by both reduced motivation to engage in mentally demanding activities and impairments in cognitive functioning (Rock, Roiser, Riedel, & Blackwell, 2014). While the causal relationship between these motivational and cognitive impairments has been a matter of controversial debate (Moritz et al., 2017; Scheurich et al., 2008), our findings inform this debate by identifying the perceived capacity to exert mental effort as a crucial precondition for deciding to engage in demanding goal‐directed behavior.

To conclude, our findings suggest a causal role of DLPFC for both exerting mental effort and informing cost–benefit decision processes about the likelihood of successful effort exertion. A computational model that takes accumulated effort into consideration revealed evidence for DLPFC involvement both in predicting the capacity for mental work and in updating this information with increasing fatigue. By showing that mental exertion converges with the computation of its costliness in DLPFC, our findings point to an efficient organizational principle of the brain.

## CONFLICT OF INTEREST

The authors declare to have no conflict of interest.

## AUTHOR CONTRIBUTIONS

Alexander Soutschek and Philippe N. Tobler designed research; Alexander Soutschek collected and analyzed data; Alexander Soutschek and Philippe N. Tobler wrote manuscript

## DATA AVAILABILITY STATEMENT

Raw behavioral data will be available online (https://osf.io/w7259/).
